# PEEP-ZEEP Compared with Bag Squeezing and Chest Compression in Mechanically Ventilated Cardiac Patients: Randomized Crossover Clinical Trial

**DOI:** 10.3390/ijerph20042824

**Published:** 2023-02-05

**Authors:** Taís Flores de Oliveira, Vinicius Serra Peringer, Luiz Alberto Forgiarini Junior, Bruna Eibel

**Affiliations:** 1 Instituto de Cardiologia, Fundação Universitária de Cardiologia (IC/FUC), 395 Princesa Isabel Avenue, Porto Alegre 90620-001, Brazil; 2 Department of Physiotherapy, Universidade La Salle (Unilasalle), Canoas 92010-000, Brazil

**Keywords:** physiotherapy, mechanical ventilation, intensive care unit, cardiopathies, respiratory aspiration

## Abstract

**Background and Objectives**: Perform the bag squeezing and PEEP-ZEEP techniques associated with manual chest compression in mechanically ventilated cardiac patients in order to observe their effectiveness in the removal of pulmonary secretions and safety from a hemodynamic and ventilatory point of view. **Methods:** This is a randomized crossover clinical trial developed in a hospital in southern Brazil. We included hemodynamically stable male and female patients aged over 18 years who used invasive mechanical ventilation for at least 48 h. The control group was established for the bag-squeezing technique and the intervention group for the PEEP-ZEEP maneuver, both associated with manual chest compression. Tracheal aspiration was performed 2 h before in order to match the groups in relation to the volume of secretion, and also immediately at the end of the techniques in order to measure the amount of secretion collected. **Results:** The sample had 36 individuals with a mean age of 70.3 years, 21% of the patients were male, and the majority (10.4%) were hospitalized for ischemic heart disease. DBP (*p* = 0.024), MAP (*p* = 0.004) and RR (*p* = 0.041) showed a significant difference in the post-moment in both groups. There was a significant difference in the reduction of peak pressure values (*p* = 0.011), in the moment after performing the techniques, and also in the Cdyn (*p* = 0.004) in the control group versus moment. **Conclusions:** Both maneuvers are safe in terms of hemodynamics and ventilatory mechanics, in addition to being capable of favoring airway clearance through secretion removal, and they can be used in routine physiotherapeutic care.

## 1. Introduction

Mechanical ventilation (MV) totally or partially replaces spontaneous breathing and is used in the cases of patients with acute or acute chronic respiratory failure in order to favor gas exchange and reduce the work of breathing [1]. Cardiac patients may manifest typical symptoms such as dyspnea, cough and fatigue during excessive exercise [2]. Many develop complications that evolve into acute cardiogenic pulmonary edema, necessitating the use of MV [3,4]. From this, physiotherapy acts with techniques aiming to reduce lung injuries and clear airways [5].

Bag squeezing is a technique performed to improve oxygenation by displacing pulmonary secretions. It is performed using a manual artificial respiration unit that is composed of a reservoir bag coupled to an oxygen flowmeter at 5 L/min [6].

The positive end-expiratory pressure-zero end-expiratory pressure maneuver (PEEP-ZEEP) considers that by raising PEEP, gas redistribution occurs through collateral ventilation, where the alveoli that are collapsed by mucus are reached. Subsequently, small airways are opened, and the adhered mucus is displaced. With the reduction of PEEP, the expiratory flow pattern is modified, causing the secretions located in smaller airways to be transported to the central airways [7].

The present study aims to evaluate the amount of aspirated secretion after performing the bag-squeezing and PEEP-ZEEP techniques associated with manual chest compression in order to observe which is the most effective in removing pulmonary secretions and safest from a hemodynamic point of view. and ventilation in cardiac patients submitted to invasive mechanical ventilation

## 2. Methods

This is a cross-randomized clinical trial developed at the Instituto de Cardiologia de Porto Alegre/RS Brazil, following the rules of the Consort Statement [8]. It is approved by the Research Ethics Committee of the Institute of Cardiology of Porto Alegre, with opinion CAAE 42228921.6.0000.5333. All those responsible for the patients signed the Free and Informed Consent Form.

The survey was carried out from February 2021 to December 2021, when all individuals were admitted to the Post-Operative Unit or Intensive Care Unit. We included hemodynamically stable male and female patients aged over 18 years who used invasive mechanical ventilation for at least 48 h. Exclusion criteria were the presence of a chest drain, subcutaneous emphysema, rib fracture, hemodynamic instability (BP < 59 mmHg or 120 mmHg), severe bronchospasm, peak pressure > 40 cmH_2_O, PEEP > 10 cmH_2_O, tracheostomy, pneumothorax and a non-drained hemothorax.

Of the 36 subjects, 31 met the inclusion criteria and were randomized by a blinded researcher, using the randomization.com program in a 1:1 crossover block, allocating the patient to one of the groups, and thus determining which of the techniques would be performed on the first day. Mucolytics were not administered in patients.

The control group was established for the bag-squeezing technique and the intervention group for the PEEP-ZEEP maneuver associated with manual chest compression. In both, tracheal aspiration was performed 2 h before in order to match the groups in relation to the volume of secretion, and also immediately after the end of the techniques in order to measure the amount of secretion collected.

Control tracheal aspiration was performed with the patient in dorsal decubitus and the headboard elevated at 30°, using a size-12 probe (Mark Med), with vacuum set at −40 cmH_2_O [9].

In the control group, patients were in the supine position, and manual and rhythmic hyperinflations were performed, alternating with manual chest compressions during expiration. Insufflation was performed slowly with a high tidal volume, followed by an inspiratory pause of two to three seconds and then the rapid release of the resuscitator. The technique was performed for 10 uninterrupted minutes [10,11].

Patients in the intervention group had their ventilatory mode adjusted to volume-cycled assist-controlled, and 6 mL/kg of predicted weight was calculated. During the maneuver, during the inspiratory phase, PEEP (positive end-expiratory pressure) was increased to 15 cmH_2_O, with an inspiratory pressure limit (PPI) of up to 40 cmH_2_O, which was maintained for 5 respiratory cycles when in the inspiratory phase. PEEP was abruptly reduced to 0 cmH_2_O, and then manual thoracic compression was associated; when starting a new inspiratory phase, PEEP was readjusted to the value initially programmed for the patient. After this first stage, we expected to perform 2 respiratory cycles, and the maneuver was performed again for 10 min [7,12].

Hemodynamic data were recorded using the multiparameter monitor at the inpatient units (Philips), and respiratory mechanics data were collected from the mechanical ventilator screen (Servo S; Drager; Newport; Leistung) before and after the techniques. The amount of aspirated secretion was deposited in the collection flask (Water Seal 120 mL) and weighed on a high-precision Ohaus AdventurerTM scale, deducting the weight of the flask.

### Statistical Analysis

In the characterization of the sample, qualitative variables were expressed through absolute and relative frequencies, while quantitative variables were expressed through mean and standard deviations or standard error. The interactions between the group and moment of hemodynamic variables and ventilatory mechanics were evaluated through the EGE, and, when significant, the Bonferroni multiple comparisons test was used as a complement. Median and interquartile ranges were adopted to analyze the amount of secretion and the non-parametric Wilcoxon Signed-Rank test was used to compare groups. The significance level was 5%. Based on a previous study (WINPEP) [+], in order to detect a minimum difference of 1.4 g in the increase in secretion removal, with an error α = 5% and a power of 80%, the minimum number of calculated subjects was 122 (61 in each group), already accounting for possible losses.

## 3. Results

**Figure 1:**
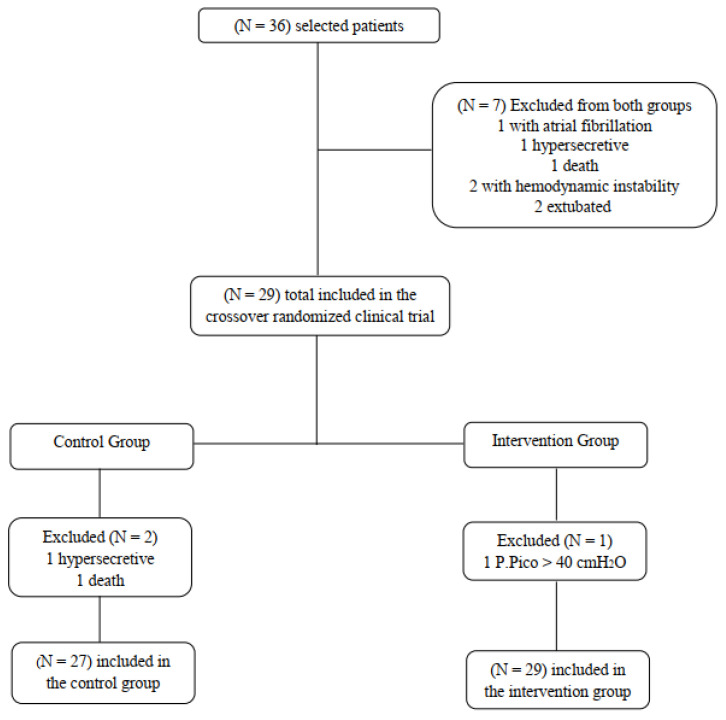
Flowchart of patients included in the study.

A total of 36 subjects were selected for the study, of which 7 were excluded (Figure 1). Most of them (21) were male, with a mean age of 70.3 ± 10.6 years. Of these patients, 12 (8.3%) had their body mass index (BMI) classified as normal and 12 (8.3%) as obese. Regarding risk factors for the development of cardiovascular diseases, 29 (20.1%) were hypertensive, and 26 (18.1%) had a diagnosis of dyslipidemia. Most individuals (10.4%) were hospitalized for ischemic heart disease, and the most prevalent associated diseases (13.9%) fell into tother category (hypothyroidism, asthma, chronic obstructive pulmonary disease, stroke, heart failure respiratory tract, anemia, hepatitis, neoplasms, sepsis and urinary tract infection). Only four (2.8%) patients underwent cardiac surgery.

During hospitalization, 11.1% had complications, such as delirium, acute myocardial infarction during surgery, seizures, ischemic intestinal ulcer, hypoxic encephalopathy, or the need for pacemaker placement and intra-aortic balloon; in addition, 14 (9.7%) patients had cardiorespiratory arrest, and 10 (6.9%) had arrhythmias. With regard to the use of medication, 17 (11.8%) needed vasoactive drugs, and 32 (22.2%) needed antibiotics (Table 1).

Regarding ventilatory therapy, the ventilatory modes used were PCV, VCV, PSV and IPPV, with PCV being the most frequent mode, with 13 (41.9%) patients in the control group and 12 (40%) in the intervention group. In addition, PEEP had a mean of 6.9 ± 1.3, while the FiO_2_ was 35.1 ± 13.2 (Table 2).

In Table 3, it is possible to observe the comparison of hemodynamic variables, where DBP (*p* = 0.024), MAP (*p* = 0.004) and RR (*p* = 0.041) showed significant results in the post-moment, having their values increased after the application of both techniques.

The comparison of ventilatory mechanics variables can be analyzed in Table 4, where a significant difference (*p* = 0.011) is demonstrated in the reduction of PPeak values at the moment after performing both techniques. In addition, the Cdyn variable also showed a statistically significant difference (*p* = 0.004) in the control group versus moment, with an increase in its value after the technique being performed in the control group. The other variables did not show significant differences.

**Figure 2:**
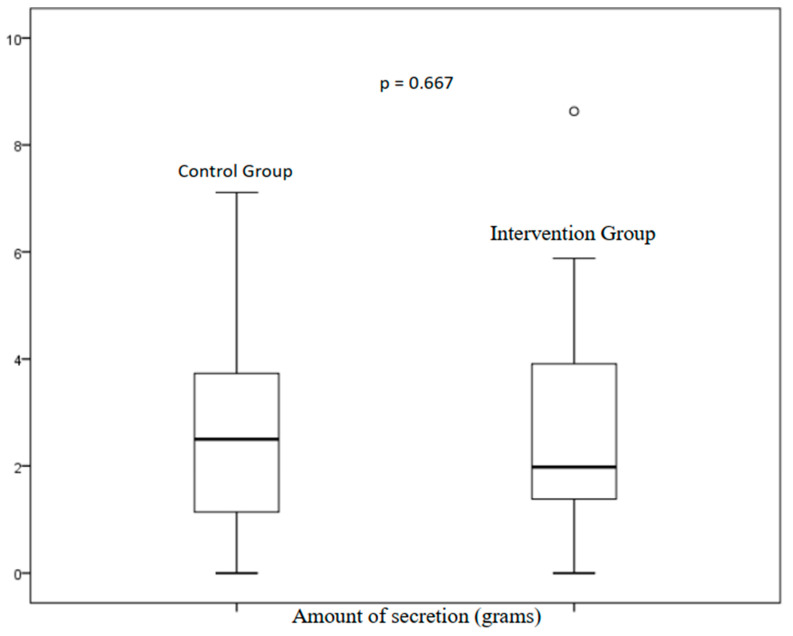
Amount of aspirated secretion between groups; median and interquartile ranges.

The amount of aspirated secretion, represented by Figure 2, is demonstrated by the median (interquartile range); the control group presented 2.4 g (1.4–3.7) compared to the intervention group, which presented 1.8 g (1.3–3.7), with no significant difference (*p* = 0.667).

## 4. Discussion

According to the analysis of the results of this study, it is possible to observe that bronchial hygiene maneuvers are important maneuvers to carry out within a physiotherapeutic service in an intensive care unit, because although there is no difference between the force that is pushing away and between the techniques performed, the strategies that are used function by mobilizing the mucus and facilitating its removal via tracheal aspiration, in a safe way and without harming the hemodynamic state of the patient [7,9,10].

As observed, most individuals have obesity, hypertension and dyslipidemia, factors that are associated with the appearance of cardiovascular diseases, as they predict the emergence of future micro- and macrovascular complications [13,14]. Such factors may be responsible for diseases such as ischemic heart disease [15], which was the most common disease among all study subjects. Although there is an increase in DBP, MAP and RR values after performing the techniques, these changes are within the normal range. In the study by Lobo et al. [10], DBP and RR also showed an increase; however, it was not significant. As in other studies that obtained similar results, we demonstrated that small changes in the values of these variables are not enough to trigger hemodynamic disorders [10,16]. When observing the PPeak values before and after performing the maneuvers, their values show a significant reduction, as in a similar result found in the study by Asmann et al., who compared mechanical hyperinflation with isolated aspiration in order to assess which was better for removing a greater amount of secretion [17]. In addition to the PPeak, the CDyn also showed a significant difference after the application of the maneuvers, a result that can be explained by pulmonary hyperinflation, which generates a better distribution of the respiratory flow and, consequently, promotes the re-expansion of the collapsed alveolar units [18]. The other analyzed ventilatory mechanics variables did not show significant results.

Even with no significant differences, both techniques prove to be effective for mucus displacement and the removal of pulmonary secretions [5]. Volpe et al. say that in order to remove secretions more effectively during bag squeezing, a slower insufflation should be performed, resulting in an adequate expiratory flow and thus displacing greater amounts of mucus [19]. In addition, manual hyperinflation associated with chest compression also seems to be efficient in improving arterial oxygenation levels, as observed by Khalil et al. [20] when analyzing the effects of the technique on arterial blood gases in patients on mechanical ventilation. On the other hand, a study that evaluated the bag-squeezing maneuver in a mechanical model of the respiratory system known as the training lung observed that the technique was not effective for the elimination of secretions, showing that even if the applicator had received instructions on how to develop the maneuver, the method was not done exactly as it should have been [21].

Similar to this study, other findings prove that the bag-squeezing and PEEP-ZEEP techniques are safe in relation to individuals’ hemodynamic and ventilatory systems, as there were no significant changes that were sufficient for their decompensation [10,16]. PEEP-ZEEP presents a good response for both the removal of pulmonary secretions and patient safety, providing an alternative, since it is not necessary to turn off the mechanical ventilator to perform it, which facilitates its application and reduces the risk of contamination when opening the suction system [16]. This research has some limitations, such as the small number of patients included; this can be explained by the COVID-19 pandemic, which generated a low demand for healthcare due to non-respiratory problems. In addition, only evaluating the variables immediately after the application of the techniques does not allow for the prolonged monitoring of patients’ hemodynamics. Furthermore, the amount of aspirated secretion may be directly related to the pathology, as patients with pulmonary infections tend to have a greater amount of mucus, and those who use antibiotics may have a reduced secretion production, which may cause bias due to changes in the amount of secretion obtained.

## 5. Conclusions

Both analyzed maneuvers are capable of promoting airway clearance through the safe removal of secretions. Therefore, we observed that the techniques can be used in day-to-day physical therapy sessions for mechanically ventilated patients with heart disease. However, PEEP-ZEEP should preferably be used, since it can be performed without disconnecting the mechanical ventilation circuit, which reduces the risk of respiratory contamination.

## Figures and Tables

**Table 1:** Sample characteristics.

Variable	N = 36 (%)
Age *	70.3 ± 10.6
Sex	
Female	15 (10.4)
Male	21 (14.6)
BMI	
Underweight	4 (2.8)
Normal	12 (8.3)
Overweight	7 (4.9)
Obesity	12 (8.3)
Risk factors	
Smoking	14 (9.7)
Hypertension	29 (20.1)
Diabetes mellitus	17 (11.8)
Dyslipidemia	26 (18.1)
Reason for hospitalization	
Ischemic heart disease	15 (10.4)
Dilated cardiomyopathy	2 (1.4)
Valve disease	6 (4.2)
PAD	3 (2.1)
Decompensated heart failure	1 (0.7)
Others	5 (3.5)
Associated diseases	
Ischemic heart disease	4 (2.8)
Kidney disease	15 (10.4)
CAD	8 (5.6)
Arrhythmias	7 (4.9)
Others	20 (13.9)
Cardiac surgery	
Total postoperative	4 (2.8)
Complications during hospitalization	
Cardiopulmonary arrest	14 (9.7)
Arrhythmias	10 (6.9)
Respiratory failure	9 (6.3)
Cardiogenic shock	5 (3.5)
APE	6 (4.2)
Others	16 (11.1)
MV days *	5.3 ± 3.1
Drug class used	
Vasoactive drug	17 (11.8)
Sedatives	15 (10.4)
Antibiotics	32 (22.2)
Anticoagulants	6 (4.2)
Others	3 (2.1)

CAD: coronary artery disease; PAD: peripheral arterial occlusive disease; APE: acute pulmonary edema; MV: mechanical ventilation; absolute and relative frequency values; * M ± SD: mean and standard deviation.

**Table 2:** Ventilation therapy.

Variable	N = 36 (%)	
VM Mode	**Control Group**	**Intervention Group**
PCV	13 (41.9)	12 (40.0)
VCV	5 (16.1)	5 (16.7)
PSV	11 (35.5)	9 (30)
IPPV	2 (6.5)	4 (13.3)
Ventilation adjustment		
PEEP *	6.9 ± 1.3	6.7 ± 1.1
FiO_2_ *	35.1 ± 13.2	35.3 ± 12.9

MV: mechanical ventilation; PCV: pressure-controlled ventilation; VCV: volume-controlled ventilation; PSV: pressure support ventilation; IPPV: intermittent positive pressure ventilation; absolute and relative frequency values; * M ± SD: mean and standard deviation.

**Table 3:** Comparison of hemodynamic variables.

	Control Group (N = 27)		Intervention Group (N = 29)		Group	Time	G*M
Variable	Before	After	Before	After	*p* Value		
SBP (mmHg) *	121 ± 3.6	129.1 ± 4.2	119.2 ± 4.7	126.2 ± 6.2	0.592	0.083	0.845
DBP (mmHg) *	64.7 ± 2.5	72.2 ± 3.8	64.7 ± 2.7	69.5 ± 3.3	0.631	0.024	0.609
MAP (mmHg) *	82 ± 2.1	90 ± 3.1	79.9 ± 2.4	87.9 ± 4	0.428	0.004	0.998
HR (bpm) *	92 ± 4.5	94.4 ± 3.6	92.8 ± 4.3	98.9 ± 3.7	0.386	0.173	0.299
SpO_2_ (%) *	97.3 ± 0.4	97.6 ± 0.4	96.5 ± 0.5	96.8 ± 0.6	0.210	0.394	0.903
RR (bpm) *	22 ± 0.8	22.4 ± 0.9	21 ± 0.7	22.8 ± 0.6	0.798	0.041	0.154

Control group: bag-squeezing maneuver; Intervention group: PEEP-ZEEP maneuver; G*M: group versus moment; SBP: systolic blood pressure; DBP: diastolic blood pressure; MAP: mean arterial pressure; HR: heart rate; SpO_2_: peripheral oxygen saturation; RR: respiratory rate; * M ± SE: mean and standard error.

**Table 4:** Comparison of ventilatory mechanics variables.

	Control Group (N = 27)		Intervention Group (N = 29)		Group	Moment	G*M
Variable	Before	After	Before	After	*p* Value		
TV *	425.19 ± 11.9	443.1 ± 14.3	410.7 ± 11.7	442.5 ± 18.9	0.384	0.052	0.479
PPeak *	20.9 ± 0.9	19.7 ± 0.9	21.4 ± 1.1	20.8 ± 1.0	0.202	0.011	0.333
P Plateau *	18.5 ± 0.9	18.9 ± 0. 8	18.3 ± 0.9	18.4 ± 0.8	0.640	0.644	0.787
DP *	11.50 ± 0.7	11.52 ± 7.6	11.4 ± 0.9	12 ± 0.8	0.701	0.518	0.531
Cst *	36.5 ± 2. 8	36.3 ± 2.6	37.3 ± 3.7	35.2 ± 3.7	0.963	0.373	0.597
Cdyn *	26.9 ± 1.9	33.8 ± 2.7	30.3 ± 3.6	29.4 ± 4.4	0.902	0.122	0.004

Control group: bag-squeezing maneuver; Intervention group: PEEP-ZEEP maneuver; G*M: group versus moment; TV: total volume; PPeak: peak pressure; PPlateau: plateau pressure; DP: drive pressure; Cst: static compliance; Cdyn: dynamic compliance; * M ± SE: mean and standard error.

## Data Availability

Not applicable.
